# Generation of a cohort of whole-torso cardiac models for assessing the utility of a novel computed shock vector efficiency metric for ICD optimisation

**DOI:** 10.1016/j.compbiomed.2019.103368

**Published:** 2019-09

**Authors:** Anne-Marie Plancke, Adam Connolly, Philip M. Gemmell, Aurel Neic, Luke C. McSpadden, John Whitaker, Mark O'Neill, Christopher A. Rinaldi, Ronak Rajani, Steven A. Niederer, Gernot Plank, Martin J. Bishop

**Affiliations:** a Department of Biomedical Engineering, School of Biomedical Engineering & Imaging Sciences, King's College London, London, UK; b Cardiovascular Imaging Department, St Thomas' Hospital, London, UK; c Institute of Biophysics, Medical University of Graz, Austria; d Abbott, Sylmar, CA, USA; e Department of Cardiology, Guy's and St Thomas' Hospitals, London, UK

**Keywords:** Defibrillation, Cardiac arrhythmias, Cardiac modelling, Implanted cardioverter defibrillators, Cardiac electrophysiogy, CT imaging

## Abstract

Implanted cardiac defibrillators (ICDs) seek to automatically detect and terminate potentially lethal ventricular arrhythmias by applying strong internal electric shocks across the heart. However, the optimisation of the specific electrode design and configurations represents an intensive area of research in the pursuit of reduced shock strengths and fewer device complications and risks. Computational whole-torso simulations play an important role in this endeavour, although knowing which specific metric should be used to assess configuration efficacy and assessing the impact of different patient anatomies and pathologies, and the corresponding effect this may have on different metrics has not been investigated. We constructed a cohort of CT-derived high-resolution whole torso-cardiac computational models, including variants of cardiomyopathies and patients with differing torso dimensions. Simulations of electric shock application between electrode configurations corresponding to transveneous (TV-ICD) and subcutaneous (S-ICD) ICDs were modelled and conventional metrics such as defibrillation threshold (DFT) and impedance computed. In addition, we computed a novel metric termed the shock vector efficiency (*η*), which quantifies the fraction of electrical energy dissipated in the heart relative to the rest of the torso. Across the cohort, S-ICD configurations showed higher DFTs and impedances than TV-ICDs, as expected, although little consistent difference was seen between healthy and cardiomyopathy variants. *η* was consistently <2% for S-ICD configurations, becoming as high as 13% for TV-ICD setups. Simulations also suggested that a total torso height of approximately 20 cm is required for convergence in *η*. Overall, *η* was seen to be approximately negatively correlated with both DFT and impedance. However, important scenarios were identified in which certain values of DFT (or impedance) were associated with a range of *η* values, and vice-versa, highlighting the heterogeneity introduced by the different torsos and pathologies modelled. In conclusion, the shock vector efficiency represents a useful additional metric to be considered alongside DFT and impedance in the optimisation of ICD electrode configurations, particularly in the context of differing torso anatomies and cardiac pathologies, which can induce significant heterogeneity in conventional metrics of ICD efficacy.

## Highlights

• Computational modelling plays an important role in optimising ICD configurations.
• Our novel metric quantifies the relative energy used by the device to defibrillate.
• It provides useful information, in conjunction with DFT and device impedance.

## 1 Introduction

Defibrillation, via the application of a strong electric field across the heart, remains the only effective treatment for a host of otherwise life-threatening cardiac arrhythmias. Implantable cardioverter-defibrillators (ICDs) are capable of both sensing the occurrence of these lethal arrhythmias and automatically delivering the appropriate electrotherapy [5,18,29]. Standard (transvenous) ICD (TV-ICD) configurations are intra-cardiac devices, which apply strong biphasic shocks between a shocking lead (coil) placed in the right ventricular (RV) cavity and the return can (the battery generator housing) placed under the pectoral muscle. As they are directly in contact with the heart, these TV-ICDs can also perform anti-tachycardia pacing from the RV electrode [28]. In some patient groups, however, such an intra-cardiac approach is often not appropriate (or required). In these patients, the use of subcutaneous ICDs (S-ICDs) has become increasingly popular, driven by the relative ease of implantation and reduced complications (lead extractions) compared to TV-ICDs [1]. In such devices, configurations usually consist of a shocking coil placed subcutaneously, to the left of the sternum, with the return can placed at the mid-axillary line, level with the heart. S-ICDs, however, typically required approximately 5 times greater shock energy to defibrillate, compared to TV-ICDs, due to the extra-cardiac lead placement.

Despite their success in terminating arrhythmic events, ICDs are far from an optimal therapy. ICDs deposit the majority of their energy in non-cardiac tissues, meaning that strong shock-strengths are required to successfully defibrillate, limiting ICD battery life and resulting in cardiac (and non-cardiac) tissue damage and impaired mechanical function [14,23]. The relatively high incidence of inappropriate shocks (approximately 10% per annum) [6] also causes significant pain and psychological issues for ICD recipients [25]. There is thus an increasing drive to develop novel ICD electrotherapy protocols and electrode configurations that can more effectively terminate lethal arrhythmias with reduced peak shock strengths and overall shock energies.

The Defibrillation Threshold (DFT) is the conventional clinical metric used to define the energy or voltage required to defibrillate a patient using a particular electrotherapy. A series of basic science and theoretical studies have linked DFT with the shock strength required to raise 95% of myocardial tissue to an extracellular potential gradient of >5 V/cm [32,33]. This concept is related to the critical mass hypothesis, that suggests that fibrillatory activity can only be sustained by a certain ‘critical mass’ of myocardium into which it can propagate. This DFT surrogate is convenient for use in computational modelling bidomain studies, which intrinsically obtain extracellular potential fields throughout the 3D volume of the myocardium [7,13,16,17,24]. However, of crucial importance in its use in assessing ICD efficacy is that it provides no information regarding how much energy is wasted in extra-cardiac tissue for a given configuration.

In this study, we present a novel metric, termed the shock vector efficiency, which computes the relative shock energy dissipated in the ventricular myocardium, compared to the rest of the torso. We evaluate this metric by constructing a cohort of CT-derived high resolution whole torso-cardiac models, the pipeline for construction of which is described in full detail, including variants of cardiomyopathies (CM) such as dilated (DCM) and hypertrophic (HCM) and patients of different physical torso dimensions. By performing realistic bidomain simulations of shock application through ICD electrodes, we demonstrate the potential of our metric at discriminating optimal shock configurations which have similar DFTs and current pathway impedances, and its utility as an additional method of quantifying defibrillation efficacy in the latest novel variants of ICD setups.

## 2 Methods & materials

### 2.1 Model creation

#### 2.1.1 Patient selection

Anonymised contrast CT scan datasets were obtained with the Siemens Somatom Definition Flash Dual Source CT, with a resolution of 0.54×0.54×1mm. Additional higher resolution cardiac scans (0.28×0.28×0.8mm were also available for these patients. From these available scans, 3 otherwise healthy patient scans were initially chosen, who were imaged to investigate symptoms of chest pain. These initial datasets were selected based on: (1) torso size; (2) presence of soft tissue scan and a heart scan with good contrast; and (3) thoracic organs with healthy anatomy. Torso size was an important consideration due to the fact that defibrillation efficacy varies with body mass index (BMI) [9]; torsos were classified as ‘Large’, ‘Medium’ and ‘Small’ (Table 1). In addition to these 3 healthy patients, 2 further patient scans were obtained from transaortic valve implantation (TAVI) recipients, of resolution 0.64×0.64×1mm. Such patients had the advantage of wide field-of-view torso high resolution CT scans, along with high resolution separate cardiac scans (0.35×0.35×0.6mm). The 2 patients selected had no specific cardiac abnormalities. Note here that the two TAVI patients included in the cohort had torso dimensions somewhere between the Medium and Large healthy patients. All patients consented for the use of their data in ethically approved research: UK Research Ethics Committee reference number 19/HRA/0502 & 15/LO/1803.

**Table 1:** Patient and torso details of the scans used for models. Torso depth and width are measured in a transverse plane at the level of the xiphoid process.

Classification	Patient details					Torso details		
	Gender	Age	Height	Weight	BMI	Width	Depth	Height
		years	cm	kg	kg * m^−2^	cm	cm	cm
Large	Male	59	177	101	32.2 (Obese)	42.0	31.0	37.5
Medium	Male	43	178	90	28.4 (Overweight)	36.9	27.5	30.5
Small	Female	57	173	60	20.0 (Healthy)	28.7	20.0	27.5
TAVI 1	Female	–	–	–	–	38.3	27.1	42.1
TAVI 2	Male	85	180	85	26.2 (Overweight)	36.1	28.6	35.7

#### 2.1.2 Image processing & analysis

**Fig. 1:**
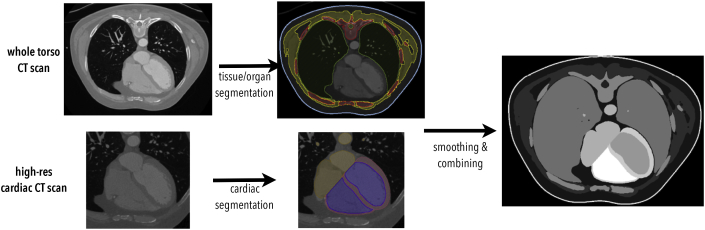
Outline of the pipeline used to generate a complete segmentation from patient-specific CT scans.

The image processing and analysis pipeline is shown schematically in Fig. 1.

Seg3D (www.sci.utah.edu/) was used to segment the CT scans of the three patients into regions of interest, based on the major organs and tissues in the torso; these included (where identifiable) the skin, skeletal muscles, fat, bones, lungs, spleen, liver, stomach, kidneys and spinal cord. The heart was segmented into separate chambers, blood pools and great vessels using the Siemens Axseg v4.11 [31] automated segmentation tool along with the higher resolution cardiac CT scans to produce smooth image segmentations, with endocardial trabeculations and papillary muscles not included. The heart segmentation was then combined with the other regions of interest in a combined segmentation of the whole torso. The CT scans for the 3 healthy patients did not encompass the entire desired extent of the torso; thus, corresponding patient MR data for the same patients was registered to the CT data and used to facilitate whole-torso model construction, allowing full representation of lungs, rib-cage and skin above and below the heart. This was not necessary for the 2 TAVI patients.

The hearts of the 3 healthy patients were then post-processed to reproduce hypertrophic cardiomyopathy (HCM, specifically, symmetrical, as in Ref. [12]) and dilated cardiomyopathy (DCM) model variants within the same overall torso geometry. HCM was modelled by dilating the left ventricular (LV) wall homogeneously to encroach on the LV blood-pool; DCM was modelled by dilation of the LV wall in the direction perpendicular to the heart's long axis, resulting in radial dilation but little dilation in the apico-basal direction. The left ventricle end diastolic diameters (LVEDD) of the original and DCM hearts, and the ventricular wall thicknesses of the original and HCM hearts, are given in Table 2; the dimensions match the values expected for healthy hearts, as well as hearts affected by HCM and DCM [19].

**Table 2:** Dimensions of the hearts used, measured in a transverse plane approximately midway up the heart, through the LV.

	LVEDD		Ventricular Wall Thickness	
	mm		cm	
	Healthy	DCM	Healthy	HCM
Large	54.7	73.4	1.0	1.9
Medium	39.8	65.0	1.0	1.7
Small	40.0	55.6	0.9	1.7

#### 2.1.3 Finite element mesh generation

**Fig. 2:**
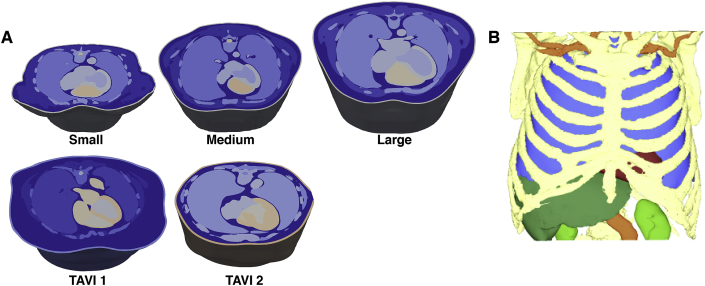
Illustrating images of 5 different meshes constituting the initial cohort. (A) Cuts through the torso, approximately along the centre line through the heart, highlighting the internal organs and tissues in each model. Images are approximately to scale, demonstrating the differences in physical geometry between patients. (B) Image of an example whole torso mesh, shown in a solid mesh format with outer skin, muscles and fat removed to allow highlighting of the lungs (blue), liver (dark green), kidneys (light green), bones (yellow), ventricles (red) and blood vessels (orange).

**Fig. 3:**
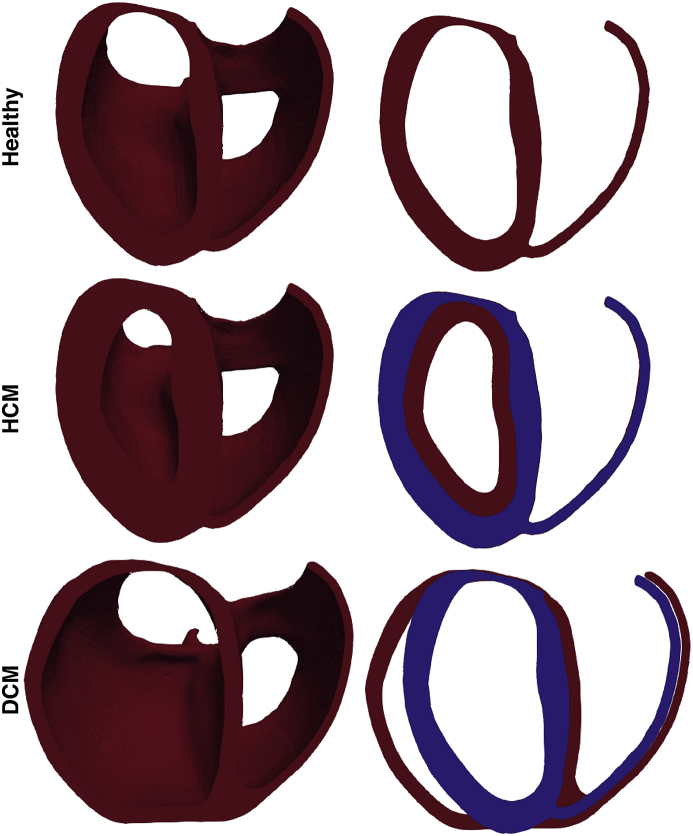
Illustrating images of the three cardiomyopathy variants modelled. Top: Healthy; Middle: HCM; Bottom: DCM. Each row shows the entire ventricles with a clipping plane used to expose endocardial surfaces (left); right images show slices taken along the same clipping plane. Blue slices correspond to the healthy case for reference.

Final combined segmentations were converted to a tetrahedral mesh using the Tarantula meshing software [21]. Segmentation tags corresponding to each organ were mapped over to individual elements as numerical tags, allowing specific functional properties to be assigned on a per region basis. In this conversion, higher resolution was used for the components of the mesh corresponding to the ventricles as the region of interest, while other regions were less refined to ensure computational tractability. Mean nodal spacing within the ventricles was approximately 600 *μ*m, with each full torso model containing approximately 75−100 million elements. Meshes were additionally smoothed on all regions using the open source software Meshtool (https://bitbucket.org/aneic/meshtool/src) due to sharp edges which may cause artefacts upon the application of strong electric fields [4]. Fig. 2A shows example images of the 5 initial whole torso models, with a transverse clipping plane used through the midline of the heart to expose the internal details. Fig. 2B shows a full torso view of the TAVI 2 patient, where the skin, fat and muscles have been rendered transparent to allow visualisation of the other internal organs. In Fig. 3, example images of the three different conditions of heart are shown for one patient, highlighting the ventricular dilation (DCM) and wall thickening (HCM) imposed within the healthy models.

#### 2.1.4 Cardiac fibre representation

Anisotropic conduction due to the myofibre architecture within ventricular myocardium is known to have important implications in the response of the tissue to strong external electric fields. In contrast to earlier whole-torso cardiac models [12,16,17], we explicitly included an anatomically-realistic representation of cardiac fibre orientation within the ventricular tissue using a previously validated approach, as described in Ref. [2], described briefly below.

Based on the interface between the segmented components of the heart, the base, epicardium and endocardium were defined, and the point apex specified manually. A smoothly varying field was then defined between the apex and the base by solving Laplace's equations with Dirichlet boundary conditions at these surfaces, and similarly for the epi- and endocardium; the gradient of these fields determines the apicobasal and transmural directions. These two directions were used to define a local coordinate system for each point in the mesh. For each point, a rotation angle *α* was calculated according to

(1) $$\alpha \left(d\right)=\alpha_{\text{endo}}\left(1-d\right)+\alpha_{\text{epi}}d,$$

where *d* is the transmural depth (normalised from 0 to 1), $\alpha_{\text{endo}}$ is the rotation angle on the endocardial surface, and $\alpha_{\text{epi}}$ is the rotation angle on the epicardial surface. The literature gives $\alpha_{\text{epi}}=-60\;\text{deg}$ and $\alpha_{\text{endo}}=60{}^{\circ}$ as acceptable values [26]. The rotation angle varies smoothly between the endo- and epicardium and defines the fibre direction.

### 2.2 Model simulation

#### 2.2.1 Tissue conductivities

Save for the heart, all other organs were assumed to be a homogeneous resistor with negligible capacitance [12]. Conductivity values were as in Ref. [12], with spinal cord conductivity from Ref. [10] and spleen conductivity from Ref. [8]. The conductivity values used are summarised in Table 3.

**Table 3:** Organ conductivity values.

Organ	Conductivity (S/m)
Blood	0.6667
Fat	0.0500
Kidney	0.1667
Liver	0.1667
Lungs	0.0714
Muscle (skeletal)	0.4444
Skin	0.0500
Spinal Cord	0.1000
Spleen	0.1000
Stomach	0.1000

Conductivities of ventricular myocardium were assigned values from the literature as 0.24, 0.035 S/m in the intracellular fibre/cross-fibre directions and 0.24, 0.2 S/m in the extracellular fibre/cross-fibre directions  [15].

#### 2.2.2 Cardiac tissue representation

In the general simulation framework, the ventricular myocardial tissue was represented by the bidomain model of cardiac electrophysiology, recast in elliptic-parabolic form [3].

(2) $$\begin{array}{c} -\nabla \cdot \left(\sigma_{i}+\sigma_{e}\right)\nabla \varphi_{e}=\nabla \cdot \sigma_{i}\nabla V_{m},\\ -\nabla \cdot \sigma_{b}\nabla \varphi_{e}=I_{eb},\\ \beta C_{m}\frac{\partial V_{m}}{\partial t}=\nabla \cdot \sigma_{i}\nabla \varphi_{i}-\beta I_{ion}\left(V_{m},\nu \right) \end{array}$$

where $\varphi_{i}$ and $\varphi_{e}$ are the intra and extra-cellular potentials, $\varphi_{b}$ is the potential of the bath (representing the surrounding non-myocardial tissues), $V_{m}=\varphi_{i}-\varphi_{e}$ is the transmembrane potential, $\sigma_{i}$ and $\sigma_{e}$ are the intra and extra-cellular conductivity tensors, β=1400 cm^−1^ is the membrane surface area to volume ratio, $I_{m}$ is the transmembrane current density, $C_{m}=1$ *μ*F/cm^2^ is the membrane capacitance per unit area and $I_{ion}$ is the membrane ionic current density, as a function of the transmembrane potential $V_{m}$ and the vector of state variables ν. At ventricular myocardium boundaries, no flux boundary conditions are imposed for $\varphi_{i}$, with $\varphi_{e}$ being continuous. At the boundaries of the conductive bath surrounding the tissue (in this case, the extremities of the torso i.e. the skin), no flux boundary conditions for $\varphi_{e}$ are imposed. Due to the nature of this specific setup, no extracellular stimuli applied to the interstitial space is present, and nor is any transmembrane stimulus. The only stimulus represented, therefore, is the extracellular stimulus applied to the bath, $I_{eb}$, defined between the ICD-electrodes. Note that all non-cardiac tissues are defined here as ‘bath’.

For simulations of DFT (using the empirical metric $\left|\left|\nabla \varphi_{e}\right|\right|>5$ V/cm in more than 95% of tissue), two setups were used for the ventricular myocardium. In the first, the tissue was assumed to behave passively, wherein the transmembrane ionic current was represented by

(3) $$I_{ion}=\frac{V_{m}}{R_{m}},$$

where $R_{m}$ is the membrane resistance with a standard value of 9 kΩ/cm^2^ and $V_{m}^{rest}=-80$ mV. Note that values of $R_{m}$, $V_{m}^{rest}$ and *β* vary and those chosen are within the range of experimentally obtained values commonly used in computational cardiac modelling [20]. This first full bidomain setup was also used to compute the total impedance of the tissue which required injection (or withdrawal) of extracellular current, $I_{eb}$, at the ICD electrodes. The second representation solved the Laplace problem, where the capacitive effects of the ventricular myocardium are ignored and the ventricular myocardium behaves as a simple (yet still anisotropic) conductor; in this case, Equation (2) simplify to

(4) $$\begin{array}{c} \nabla \cdot \left(\sigma_{i}+\sigma_{e}\right)\nabla \varphi_{e}=0,\\ \nabla \cdot \sigma_{b}\nabla \varphi_{e}=0, \end{array}$$

where the top equation is solved in the (anistropic) ventricular myocardium and the lower equation in all other non-ventricular (isotroptic) tissues and organs which were treated as extracellular conductors (the bath). Appropriate boundary conditions are applied to nodes representing the electrodes defining the ICD setups being 1 for positive (shocking) coils and 0 for negative ground electrodes.

#### 2.2.3 ICD electrode modelling

TV-ICD and S-ICD setups were modelled. Two variants of TV-ICD configurations were represented, including a 5 cm long shocking electrode inside the right ventricle (RV) (**TV-ICD1**) with a sub-clavicle can, with a further representation including an additional 8 cm long ground electrode in the Superior Vena Cava (SVC) (**TV-ICD2**). In the latter case, two shock vectors are simultaneously used, one between the RV coil and can, and the other between the RV coil and SVC coil. For the standard S-ICD representation, the subcutaneous shocking electrodes were defined as cylinders of 8 cm in length and 3 mm diameter. The can was defined as a cylinder of 7 cm diameter and 2 cm thickness, placed at the mid-axillary line at the level of the 6 th and 7 th ribs, within muscle and fat tissues with approximate respective mesh discretisations of 1.1 mm and 0.6 mm, respectively, thus faithfully resolving the can morphology. It is noted, however, that more recent devices may be of slightly slimmer design. During simulations, boundary conditions were specified on all finite element nodes within the geometrical regions defined by the electrodes above. For shocking electrodes, extracellular potential was fixed at the specified values to define the strength of the applied shock; for ground electrodes, extracellular potential was defined to be 0 mV throughout.

#### 2.2.4 Simulation protocols

When using the full bidomain representation of cardiac tissue, monophasic shocks were applied by delivering a constant voltage across shocking electrodes for a duration of 30 ms. As the time constant of the membrane is approximately 5 ms, delivering a 30 ms shock ensured approximate steady-state of the system had been reached. In the case of using a simple Laplace solve, a single static solution was obtained due to its temporal independence.

For each electrode configuration, the impedance (resistance) of the pathway between the electrodes was also computed. To this end, simulations of a constant current injection between shocking and ground electrodes were performed with membrane dynamics set to be passive.

### 2.3 Data analysis

#### 2.3.1 DFT computation

Extracellular potentials throughout the ventricular myocardium at shock-end were used to evaluate DFT. Specifically, in accordance with previous experimental [32,33] and simulation [7,13,16,17,24] studies, the voltage level at which 95% of the ventricular myocardium had $\left\|\nabla \varphi_{e}\right\|>5$ V/cm was defined as the DFT. A defined voltage of 50 V was applied to all models, and then linearly scaled to define the DFT at which the above criteria were met. A test to check for linearity was performed whereby the applied voltage was increased by a factor of two and the DFT re-computed.

Different possible methods exist to compute the energy delivered by the capacitor. We chose to compute the stored energy in terms of the capacitance by $\text{Energy}=\frac{CV^{2}}{2}$, in-line with previous simulation studies [16], where C=100 *μ*F and *V* is the required voltage DFT for the particular electrode configuration.

#### 2.3.2 Impedance computation

To compute impedances (resistances), simulations of total current injection were used and average potentials were extracted over the surface of the shocking electrodes to compute voltage-drops between the shocking electrode and the ground electrode. The specific resistances of the electrode configurations was then derived via Ohm's Law, R=V/I.

#### 2.3.3 Shock vector efficiency

In addition to quantifying the efficacy of a particular electrode setup in terms of the DFT (as a voltage or equivalent stored energy), we also introduce here the quantification of a metric which we term the *shock vector efficiency*. The shock vector efficiency (*η*) represents the ratio of the electrical energy which is dissipated (via Joule heating) within the ventricular myocardium, relative to the total electrical energy dissipated within all other tissues and organs within the torso (including the myocardium).

If the power dissipated (*P*) *per unit volume* (*τ*) is

(5) P=J⋅E

where J is the current density and E the electric field within the tissue and J=σE, where σ is the tissue-specific electrical conductivity. For all of the non-ventricular myocardial tissues and organs within the torso, the tissue is assumed to be isotropically-conducting, meaning that the conductivity tensor σ becomes a scalar and J and E are in the same direction. For ventricular myocardial tissue, conductivity is defined to be anisotropic, and thus the full tensor form of σ must be used, constructed from the fibre orientation data defined in Section 2.1.4.

As electrical power and energy are equivalent here for a fixed duration shock, we thus define the shock vector efficiency to be

(6) $$\eta =\frac{\int_{\tau_{myo}}J\cdot Ed\tau }{\int_{\tau_{total}}J\cdot Ed\tau },$$

where $\tau_{total}=\tau_{myo}+\tau_{non-myo}$. *η* thus gives a representation of the fraction of the shock's energy that is actually delivered to the ventricles, relative to that delivered to the whole torso.

It is noted here that we applied fixed strength shocks, not biphasic shocks with exponentially decaying tilts. However, as all impedances are represented as Ohmic, as voltage changes with time, the same fraction of voltage is dropped across the heart, relative to the rest of the torso. Thus, ignoring capacitive effects and treating the torso and heart as a simple Ohmic resistor, the shock vector efficiency would remain constant throughout the biphasic shock. We do not, however, explicitly compute the total energy delivered by the device. Consequently, the metric is only a comparison of relative energies between the myocardial and whole torso domains; it does not represent the explicit computation of the energy dissipated by the device as we do not simulate the full nature of a biphasic stimulus pulse, as noted.

### 2.4 Comparison of simulation methods

Full bidomain solutions (considering the capacitive properties of the heart, solving the full equations as stated in Section 2.2.2 with a passive cell model) in addition to simple Laplace solutions (solving only the middle equation in Section 2.2.2) were computed initially for all healthy torso models in the cohort. Differences in computed DFTs were seen to be consistently less than 2% using the $\varphi_{e}$ solutions taken at shock-end in the bidomain solves (mean values of initial cohort of 5 patients) in comparison to the Laplace solutions. Thus, throughout the rest of the study, simple Laplace solves were performed for $\varphi_{e}$ simulation and obtaining DFTs. Full bidomain simulations were performed for impedance calculations, as described in Section 2.2.2.

## 3 Results

### 3.1 Defibrillation threshold

**Fig. 4:**
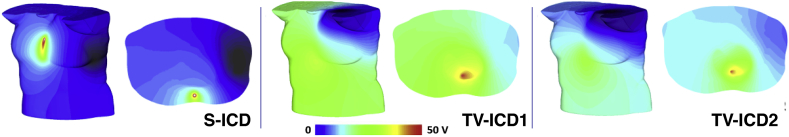
Distribution of $\varphi_{e}$ throughout the torso volume for each different electrode configuration for a stimulus of 50 V. Each panel shows frontal views (left) as well as views with a clipping plane exposing the intra-torso cavity along the mid-line (right). Example torso corresponds to TAVI 1 model.

Fig. 4 compares the distribution of $\varphi_{e}$ throughout the torso volume for the subcutaneous and two tranveneous electrode setups. A number of important features are demonstrated in this figure; firstly, the distribution of $\varphi_{e}$ throughout the whole torso is very different for each configuration; and, secondly, that the rate of decay (i.e. the gradient) of the potential is also very different throughout the heart.

**Fig. 5:**
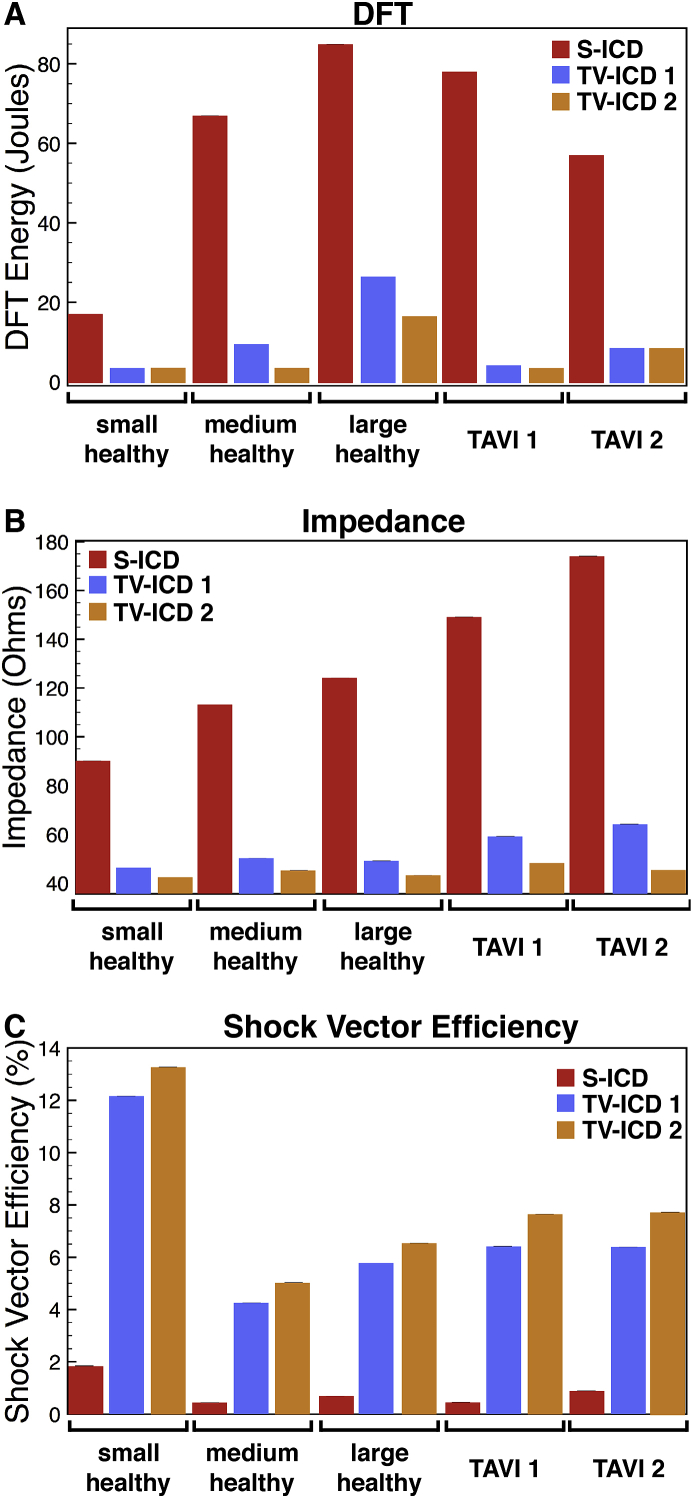
DFTs (panel A, in energy), electrical impedance (panel B), and shock vector efficiency (panel C) shown for each patient in the cohort, comparing different electrode configurations (coloured bars).

Quantification of the DFT accounts for these differences in the gradient of $\varphi_{e}$ throughout the heart. Fig. 5A presents the energy DFT for all torsos, comparing different electrode configurations. Here, it is clear that the different electrode configurations result in substantially different DFTs for each torso, along with the expected difference in DFT for different configurations within a single torso. Specifically, the values for the S-ICD configuration are substantially higher (generally, over 5 times higher) than the transvenous configurations, which are generally <10 J, as expected. Also, as expected [27], DFTs of single coil devices are slightly higher than dual-coil devices.

### 3.2 Electrical impedance

The specific DFT within each model is sensitive to the volts ‘dropped’ across the heart, which is governed by the organ anatomy and their respective conductivities. Measurement of impedance (resistance) in this case can provide a useful indication of the reasons for differing DFTs between models, in relation to different specific current pathways. For each electrode setup, electrical impedance was computed between anode and cathode electrodes. Fig. 5B details all impedance values for all models in the cohort, for all electrode placements.

In general, the smaller torsos have lower impedances for all electrode configurations, compared to the larger torsos, as expected due to the closer physical proximity of electrodes in these cases. All transvenous configurations also result in substantially lower impedances across the cohort; again expected due to the shorter current paths and the less high-resistance tissue (e.g. bones, fat) the current must transverse between electrodes.

### 3.3 Shock vector efficiency

Fig. 5C shows the shock vector efficiency computed from $\varphi_{e}$ for each patient in the initial cohort for all electrode configurations. Immediately, a significant difference between S-ICD and TV-ICD configurations is evident, with *η* for TV-ICD configurations typically being 5 times greater than *η* for the S-ICD configuration. There is also a consistently higher *η* for the TV-ICD 2 configuration (including the extra SVC electrode), relative to the TV-ICD 1 configuration. The Small torso also has a noticeably larger *η* value across the configurations compared to other patients. However, the Large torso has a higher *η* than the Medium torso, indicating that *η* does not necessarily scale with torso size.

### 3.4 Convergence of *η* with torso dimensions

As seen in Equation (6), the computation of *η* explicitly depends upon the volume of torso included in the calculation. Thus, we assessed how the computed value of *η* depends on the size of the torso modelled; only the S-ICD setup was considered as the TV-ICD required larger torso sizes due to their higher electrode placements. This was done in two separate ways: firstly, the simulation of an applied shock was performed on a full size torso model (the TAVI 2 model), but only progressively larger regions of torso either side of the heart were included in the post-simulation analysis (in Equation (6)); secondly, torsos of differing dimensions (including progressively larger regions of torso either side of the heart) were used in the initial simulation of the applied shock (and for subsequent analysis).

**Fig. 6:**
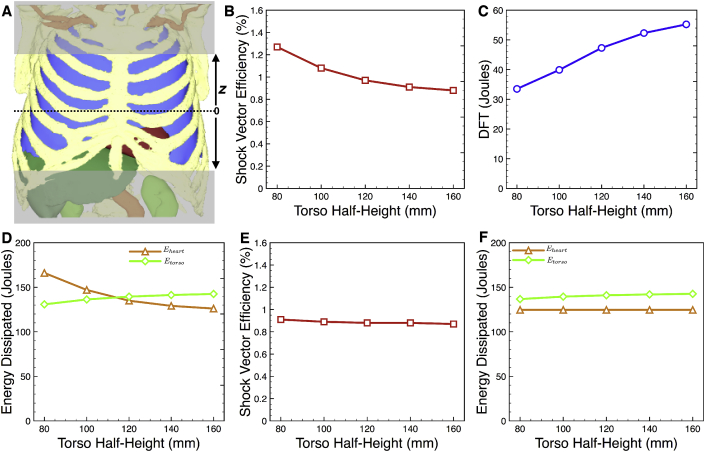
Convergence of η (and DFT) for different size torso. A) Schematic representation of how differing heights of the torso were excluded from the simulation and/or analysis; half-torso height is defined as the distance from the mid-point in the axial direction. B-D) Variation of η, DFT and energy dissipated within the heart and torso with half-torso height incorporated in both the simulation and analysis; E-F) Variation of η and energy dissipated within the heart and torso with half-torso height incorporated in only the analysis, where simulation was performed on the entire torso volume. Simulation data shown is for the TV-ICD setup in the TAVI 2 patient model.

Fig. 6B plots the value of *η* as a function of the half-torso height included in the torso model as the half-height increases from 80 to 160 mm (shown schematically in Fig. 6A), along with the DFT for comparison in Fig. 6C. Whilst the DFT increases as the size of the torso modelled increases, the shock vector efficiency decreases, although to a lesser degree. The magnitudes of these changes are important to note: DFT increases by over 60% whilst *η* decreases by approximately 30%. Fig. 6D also plots the total energy dissipated within the heart and within the torso separately. The energy dissipated within the heart monotonically decreases with torso size (by approximately 25%), whereas the energy dissipated within the torso increases slightly (by less than 10%).

Finally, in Fig. 6E–F, similar data is shown to panels B & D, respectively, however, in this particular case simulations were conducted with the entire torso (with normal conductivity), but analysis of *η* was performed with the limited torso dimensions. Thus, the form of the solution (the potential and its gradient) does not change with torso height. DFT data is not shown, as the value is constant at 55.1 J, corresponding to the same value shown in Fig. 5A. In addition to a constant DFT, the energy dissipated in the heart remains constant for all torso heights considered in the analysis (as the form of the solution remains constant due to the fact that the full torso was used in its computation). Thus, the only thing to change is the total energy dissipated in the torso (the denominator in Equation (6)), which monotonically increases by approximately 8%, which therefore monotonically decreases *η*, as seen. Consequently, *η* varies very slightly as a torso height included in the post-simulation analysis changes.

### 3.5 Effect of cardiomyopathies

The extent to which pathological hearts, representing different forms of cardiomyopathies (specifically HCM and DCM) may introduce changes in DFT, electrical impedance and shock vector efficiency for each electrode configuration was then assessed. Shock simulations were repeated in the 3 healthy models within the cohort with their hearts morphologically altered to represent DCM and HCM conditions, as described in Section 2.1.2.

**Fig. 7:**
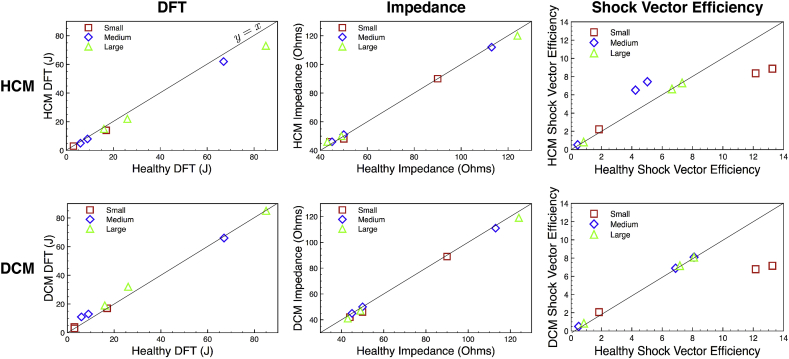
Comparison of the effect on DFTs (left), electrical impedance (centre) and shock vector efficiency (right) of HCM (upper row) and DCM (lower row) cardiomyopathies. For each metric the value is plotted in the diseased case (HCM or DCM) against the corresponding value of the metric in the healthy heart case (with no adjustments to the heart). The line of y=x is plotted to assist in highlighting differences between healthy and diseased hearts. Data for all electrode configurations are shown for Small (red squares), Medium (blue diamonds)& Large (green triangles) torsos.

Despite the significant changes in wall thickness (HCM) and cavity volume/wall thinning (fDCM) represented by these additional models (as shown in Fig. 3), little difference was generally seen in the resulting DFTs for both cardiomyopathies considered. Fig. 7 directly compares DFTs (left), impedances (centre) and shock vector efficiencies (right) between HCM and the healthy equivalent model (upper panels), along with the same for DCM (lower panels), for all electrode configurations, for each of the three patients (represented by colours). The majority of points in both panels lie on the line of y=x; however, some slight trends are seen.

Specifically, in the case of DCM, the TV-ICD setups (which can be identified as those with relatively low DFTs from comparison with Fig. 5A) tend to produce slightly higher DFTs compared to the healthy cases (lying above the line). Likewise, TV-ICD electrical impedances are marginally lower in the DCM patients. In the case of DFTs in HCM, there is a trend for a slight reduction in DFT in HCM patients; this is marginally more apparent for the S-ICD configurations. Likewise, TV-ICD electrical impedances also show a slight increase, relative to the healthy models.

Despite these marginal differences seen in DFTs and impedances between healthy and diseased hearts, more pronounced differences are seen in shock vector efficiency. Most noticeably, for example, the TV-ICD setups show a decrease in *η* in both HCM and DCM for the Small patient, whereas a small increase is seen in the Medium patient for HCM. The Small patient data appears to be an outlier in this case; on the whole, there seems to be a slight trend for increases in shock vector efficiency in both HCM and DCM across all electrode configurations.

### 3.6 Relating *η* to DFTs and impedances

**Fig. 8:**
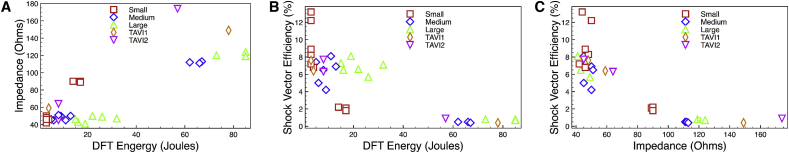
Correlation plots of DFT *versus* impedance (panel A), DFT *versus* shock vector efficiency (panel B) and impedance *versus* shock vector efficiency (panel C). Plots contain data for both S-ICD and TV-ICD configurations for all five initial patients (Small, red squares; Medium, blue diamonds; Large, green triangles; TAVI1, orange diamonds; TAVI2, magenta inverted triangles) as well as the additional cardiomyopathy variants for the Small, Medium and Large patients.

The degree to which the metrics DFT, impedance and shock vector efficiency are related to one-another is now explicitly explored by producing correlation plots. Fig. 8 shows such correlation plots, which include all simulation data for all configurations (S-ICD and TV-ICD) and all patients in the cohort (healthy, HCM/DCM equivalent, and TAVI patients) of DFT versus impedance (panel A), DFT versus *η* (panel B), and impedance versus *η* (panel C).

Although some outliers exist, Fig. 8 A suggests that DFT and electrical impedance are approximately correlated, as might be expected, such that a low (high) impedance corresponds to a low (high) DFT, with a correlation coefficient of 0.855. Furthermore, Fig. 8 B & C suggest that DFT and impedance are both negatively correlated with *η*, such that a low (high) DFT or impedance generally corresponds to a high (low) *η*, with correlation coefficients of −0.791 and −0.839, respectively. However, the plots also highlight crowding of points along both *x* and *y* directions. For example, in panel B, for lower DFTs, a number of points are seen to have very similar DFT (approximately <5 J), but have very different values of *η* (approximately 6−13%); conversely, for lower values of *η*, a number of points are seen to have very similar *η* (approximately <1%), but have very different values of DFT (between approximately 55−95 J). A similar situation is also seen in panel C; for lower values of impedance, a number of points have very similar impedances values (between approximately 40−50 Ω), but have very different values of *η* (between approximately 4−13%); conversely, for lower values of *η*, a number of points are seen to have very similar *η* (approximately <1%), but have very different impedances (between approximately 110−175 Ω).

## 4 Discussion

In this study, we present the generation of the first cohort of whole torso-cardiac models from high-resolution CT data, and incorporating all visible organ types from the imaging data, as well as anatomically-based representation of the anisotropic conduction within the heart. Simulation of shocks applied via different ICD configurations are used to demonstrate the utility of a novel shock vector efficiency metric, which is shown to be complementary to conventional metrics such as DFT surrogate (based on $\nabla \varphi_{e}$ criteria) and tissue impedance. The inclusion of a range of body sizes and compositions, along with pathological morphological alterations to the cohort, allowed us to demonstrate the key additional information provided by the shock vector efficiency that might help optimise electrode configuration design. Significant differences in all quantitative metrics analysed were seen across the cohort, emphasising the need to represent heterogeneity in patient torso and cardiac anatomy when assessing novel ICD electrode configuration efficacy.

### 4.1 Use of *η* along with other metrics

An important part of this present study was the introduction of a novel computational metric which allowed us to quantitatively compare the fraction of energy dissipated within the ventricular myocardium, relative to the entire torso, for a particular electrode configuration, which we termed the *shock vector efficiency*, *η*. In this context, the term ‘vector’ was used in-line with the standard ICD device convention [11] to describe a specific electrode anode/cathode shocking configuration (although rigorously should be reserved for a single point anode/cathode setup.) Such a metric may have important implications in future device design as maximising energy efficiency of an ICD (*for the same DFT*) would minimise the energy dissipated in the surrounding torso, potentially limiting extra-cardiac tissue damage and associated problems.

DFT is an important metric for quantifying and comparing ICD configuration efficacies, but has its limitations. For example, it does not consider current paths through other organs, only what is happening to the heart. The correlation between DFT and impedance is also not necessarily robust; generally, a low impedance will give a low DFT.

For example, the impedance of the dual-coil (TV-ICD2) is lower than that of the single-coil configuration (TV-ICD1) as the current is more ‘spread-out’ in the former increasing the effective cross-sectional area through which it flows and correspondingly decreasing the DFT slightly (Fig. 5), as expected from clinical studies [27]. Furthermore, the most obvious case of this is for electrodes which are close together, spanning the heart; here, the impedance is low (due to a short current path-length), and correspondingly the voltage gradient (across the heart) is high (as the majority of the voltage is dropped across the heart), However, a low impedance is also formed if the electrodes are close to one another but do not span the heart (as current path-length is still small), but due to the lack of field across the heart, voltage gradient within the heart is low, and so DFT is necessarily high. This highlights the potential limitations of also considering impedance (which is a clinically-measurable quantity) in its relation to DFT and/or shock vector efficacy.

A strong correlation was also seen between *η* and DFT, which perhaps may be expected as both are driven by high voltage gradients within the heart. However, the utility of our novel metric in determining the efficacy of specific electrode configurations is perhaps highlighted by the cases in which this correlation is not so strong. For example, for some values of *η*, it was seen that similar energy is needed to produce very different DFTs, but also that similar DFTs can result from very different values of *η*. Perhaps considering both *η* and DFT could be of significant utility in determining optimal ICD electrode configurations. Evidently, the DFT is the metric that determines the actual energy utilised by the device itself. However, a device configuration that has a high value of *η*, for a given DFT, would deposit more of this energy in the myocardium, where it is needed, limiting the extra-cardiac field and associated problems. This would seem to be an important future device design consideration.

Overall, the correlation plots of Fig. 8 highlight that the specific relations between DFT, impedance and *η* are complex, and depend strongly on the individual heart/torso make-up and exact nature of the current path defined by the shock vector. This emphasises the utility of considering an additional metric, such as *η*, in the optimisation of electrode configurations and shock vectors, particularly in light of the significant variation in all metrics considered within the our heterogeneous torso model cohort.

### 4.2 Variation within cohort

In general, the specific distribution of voltage within the heart for a given shock strength and electrode configuration (which in turn govens DFT, impedance and *η*) is determined by two factors: 1) the physical separation (and location) of the electrodes defining the configuration and shock vector; and, 2) the exact constitution of organs in a particular patient, and where they specifically lie with respect to the applied shock vector and resulting current paths.

In smaller torsos, the reduced distance between the electrodes leads to the production of a stronger electric field, and hence overall higher voltage gradients, meaning that lower DFTs are required. For the majority of configurations, the Small torso had the lowest DFT, then the Medium, with the Largest patient having the biggest DFT. Furthermore, the impedances of the configurations were seen to be generally higher as the size of the torso increased. The slightly heterogeneous nature of the DFTs from these patients is possibly explained by the presence of the lung, extending down in front of the heart for TAVI 1 patient, and by a high amount of fat.

This finding further emphasises that fact that due to the heterogeneous nature of the conductivities of the respective organ types, what lies within the current paths also is a key determinant of the shock efficacy metrics considered. As can be seen from Table 1 and Fig. 2, and the quantitative results shown later, patients who have an overall torso with similar dimensions can have vastly different DFT, *η* and impedances. This result emphasises the importance of assessing novel ICD configurations within a *cohort* of individuals with a range of physical torso sizes as well as body tissue composition, as performed in this work. Although there was similarity in overall trends and observations between individuals within the cohort, we believe that demonstrating these similarities represents an important aspect of this work which would not be known from a single model investigation.

### 4.3 Variation with pathology

The amount of ventricular myocardial tissue encompassed by any given shock vector is potentially important when considering different ICD electrode configurations. The effect of different cardiomyopathies, specifically HCM and DCM, which introduce distinctive changes in ventricular cavity dimensions and wall thickness, were therefore considered, in the context of changes in the quantitative metrics which relate to ICD shock efficacy of different setups. In this study, a distinct advantage was that we were able to morphologically alter the hearts within the healthy patients in the cohort, allowing us to directly compare the effects of HCM and DCM in hearts that were within exactly the same torso geometry, for 3 separate individuals. Similarly to previous studies [12], despite fairly significant changes in cardiac anatomy when representing the HCM and DCM hearts (seen in Fig. 3), there was no clear difference seen in DFTs between these different pathologies. However, although not significant, a slight trend was seen for a marginally increased DFT in the context of DCM. We believe that this is a result of the dilated heart walls moving further away from the primary shock vector, and into regions of lower voltage gradient, thus requiring a higher DFT. However, this trend was only seen in some of the patients and for some of the electrode configurations tested. Indeed some of the patients showed very slight decreases in DFT during DCM for some configurations. In the case of HCM, again, although not a significant change, there was a slight trend noticed for a reduction in DFT in HCM patients. Such a slight reduction in HCM patients was also noted in another recent study [12]. It should be noted, though, that here we consider only the geometrical effects of DCM/HCM changes; we do not represent any form of electrophysiological and tissue microstructural changes that may occur in these pathological conditions.

In addition to changes in DFT, analysing the corresponding changes in *η* also provided useful in these pathological scenarios. Specifically, a slight increase in *η* was witnessed in both HCM and DCM, relative to the healthy cases. In such pathological scenarios, myocardial tissue volume is increased, meaning that the heart occupies more of the torso volume. For example, the average increase in myocardial tissue volume was some 42% in the HCM case whilst just 2% in the DCM case. Even in the case in which the voltage field is unchanged, this would necessarily lead to an increase in *η* as more energy will be stored in the heart and less in the torso. However, it is important to realise that the electric field is highly non-linear in its decay away from the electrodes, meaning that only adding myocardial volume in the regions of highest electric field would have appreciable changes in *η*, not to mention the fact specific voltage field distributions would also differ between pathologies. Nonetheless, we believe that *η* represents an important additional metric to be considered in these cases.

### 4.4 Consideration of torso height

When performing image-based computational modelling studies aimed at quantifying electric field distributions generated by different ICDs, an important consideration is the size (or height) of torso needed to faithfully represent the specific electrode configurations under investigation. By excluding progressively larger regions from the top/bottom of one of our models we demonstrated a significant impact on the metrics used to quantify defibrillation efficacy. With larger volumes of torso included in the model, current is not as confined to flow directly through the heart itself, as the pathways for the current to flow through open up, decreasing the impedance between electrodes. This ‘spreading-out’ of the current, means that the electric field lines also spread-out, decreasing the voltage gradient through the heart, leading to a stronger applied field strength required to defibrillate, increasing DFT, as shown in Fig. 6C.

However, in the case of *η* the relationship is not as clear. Here, the decrease in field strength throughout the torso with increasing torso height means that the energy density dissipated in all organs decreases. As the volume of the heart remains constant, the numerator in Equation (6) decreases monotonically; however, in the denominator, the energy density in each organ decreases, but the volume represented by the organs (more specifically, the additional organs included as the torso increases in height) increases. It is a consequence of this that the change in *η* of the torso volumes considered is less than that of the DFT (*η* decreases by approximately 30% between torso half-heights of 80–160 mm, compared to DFT which increases by approximately 65% over the same range). Thus, we conclude that shock vector efficiency may be an important additional metric for use in comparison between different torsos within a cohort in a scenario in which the height or volume of the torsos may vary. It is also of relevance for guidance of future torso-heart model construction endeavours that the shock vector efficiency somewhat converges for torso heights of approximately >20 cm; interestingly, although DFT is also seen to converge with torso height, this seems to be less rapid than *η* e.g. between torso half-heights of 140–160 mm, *η* changes by approximately 3% compared to DFT which changes by approximately 6%.

### 4.5 Relation with critical mass hypothesis

The use of the 95% of tissue with voltage gradient >5 V/cm as a surrogate for DFT is widely used in both experimental and computational modelling fields. This criteria is based on the sound theoretical reasoning that it is the electric field strength (and its gradient) that drives the changes in membrane potential. Such changes in $V_{m}$ are key in prolonging tissue refractoriness and/or producing additional excitations, both of which are required to eliminate excitable gaps within the majority of the myocardium to terminate fibrillatory activity. More specifically, we note that these changes are driven by the combination of the electric field strength weighted by the heterogeneity in tissue conductivity, along with the gradient of the field weighted by the conductivity itself [30].

However, a recent modelling study suggested that this surrogate did not compare well to the actual shock voltage required to defibrillate simulated episodes of VT/VF [22]; although, a more recent simulation study suggested a closer correlation, comparing actual recorded clinical DFTs with voltage gradients simulated in patient-specific models (although not simulating episodes of VT/VF as in Ref. [22]). In the clinic, defibrillation is not effective 100% of the time, even when shocks may successfully achieve the $\nabla \varphi_{e}$ criterion i.e. achieving 95% of tissue with voltage gradient >5 V/cm. Thorough testing of a given configuration to reliably characterise DFT would thus require large numbers of different episodes to be simulated, each with their own dynamics as well as shocks being applied at differing phases within each episode, in order to achieve statistical significance. Furthermore, the successful defibrillation of a simulated episode of VT/VF is hugely dependant upon numerous functional parameters incorporated into the model, such as conduction velocity and ion channel kinetics, their individual restitution and heterogeneity throughout the heart. Thus, the use of more simple metrics, such as directly computing the response of the tissue to the shock via the $\nabla \varphi_{e}$ criterion, would seem a practical way of directly comparing shock efficacy of different ICD configurations i.e. the direct effect of the particular shock vector on the heart. Moreover, the use of metrics such as the shock vector efficiency presented here have the further advantage of providing additional information which may be used to compare different shock vectors.

### 4.6 Study limitations

The specific values of conductivities used for the various organ types having widely differing values reported in the literature. Variation in these values impacts the potential distribution within the heart-torso and thus quantitatively affects the metrics computed in this work (DFT, impedance, *η*). Due to this significant variation, we considered our quantitative results in the context of a single set of conductivity values, as used in a recent S-ICD modelling study [12]. We acknowledge that are simulations were performed using a simple Laplace solve and direct computation of the DFT via the $\left\|\nabla \varphi_{e}\right\|$ criteria, as described, and that we did not perform full bidomain simulations of the biphasic shock nor corresponding computations of the exact energy used by the device. Nonetheless, due to similarities seen in $\varphi_{e}$ distributions using Laplace solves and monophasic bidomain shocks (as described in Section 2.4), we believe that our results provide a useful means of directly comparing different metrics related to ICD electrode configurations in individuals.

## 5 Conclusions

The shock vector efficiency represents a useful additional metric to be considered in the optimisation of ICD electrode configurations. It may provide particularly useful information in the context of differing torso anatomies and cardiac pathologies, which can lead to significant heterogeneity in conventional metrics such as DFT and impedance. This heterogeneity between subjects also emphasises the need to conduct such simulation and practical investigations in the context of a cohort of detailed whole torso-cardiac models.

## Discloser

No disclosers.
